# Usefulness and capability of three-dimensional, full high-definition movies for surgical education

**DOI:** 10.1186/s40902-017-0107-3

**Published:** 2017-04-05

**Authors:** M. Takano, K. Kasahara, K. Sugahara, A. Watanabe, S. Yoshida, T. Shibahara

**Affiliations:** 1 Department of Oral and Maxillaofacial Surgery, Tokyo Dental College, 101-0061, 2-9-18 Misakicho, Chiyoda-ku, Tokyo, Japan; 2 Department of Oral Pathobiological Science and Surgery, Tokyo Dental College, Mihama-ku, Japan

**Keywords:** 3D movie, Medical education, Surgical practice

## Abstract

**Background:**

Because of changing surgical procedures in the fields of oral and maxillofacial surgery, new methods for surgical education are needed and could include recent advances in digital technology. Many doctors have attempted to use digital technology as educational tools for surgical training, and movies have played an important role in these attempts. We have been using a 3D full high-definition (full-HD) camcorder to record movies of intra-oral surgeries.

**Method:**

The subjects were medical students and doctors receiving surgical training who did not have actual surgical experience (*n* = 67). Participants watched an 8-min, 2D movie of orthognathic surgery and subsequently watched the 3D version. After watching the 3D movie, participants were asked to complete a questionnaire.

**Result:**

A lot of participants (84%) felt a 3D movie excellent or good and answered that the advantages of a 3D movie were their appearance of solidity or realism. Almost all participants (99%) answered that 3D movies were quite useful or useful for medical practice.

**Conclusions:**

Three-dimensional full-HD movies have the potential to improve the quality of medical education and clinical practice in oral and maxillofacial surgery.

## Background

Medical education and surgical training are indispensable for oral and maxillofacial surgeons. However, operating room restrictions can make surgical training difficult. Traditionally, medical education was a personal endeavor, and surgical techniques were passed from mentor to student by strict apprenticeships. While this tradition is not completely outdated, the attitudes of today’s young trainees and residents towards this type of training are changing.

Surgical procedures in the fields of oral and maxillofacial surgery are also changing. Consequently, new methods for surgical education are needed and could include recent advances in digital technology, such as two-dimensional (2D) and three-dimensional (3D) imaging, real-sized 3D modeling, and virtual reality systems [1–4].

Recently, many doctors have attempted to use digital technology as educational tools for surgical training of young doctors [5, 6], and movies have played an important role in these attempts. Movies have been used to show surgical operations and medical treatments, as materials for lectures and conferences and as learning tools. Medical records with an accompanying movie have also been used to explain a procedure to a patient or disclose information about a diagnosis [7, 8]. The quality of the movies has advanced, with full high-definition (full-HD) images that have approximately 2 megapixels now available. These full-HD images have twice the number of pixels of a regular HD image and up to 6 times the standard definition. With recent technical developments, 3D images are now being used for entertainment and amusement, such as in movies or television programs, and have attracted attention in medical fields because of their 3D impression, realism, and dynamics [9, 10].

We have been using a 3D full-HD camcorder to record movies of intra-oral surgeries. The purpose of this study is to assess the usefulness and capability of 3D full-HD movies for surgical education.

## Methods

**Fig. 1:**
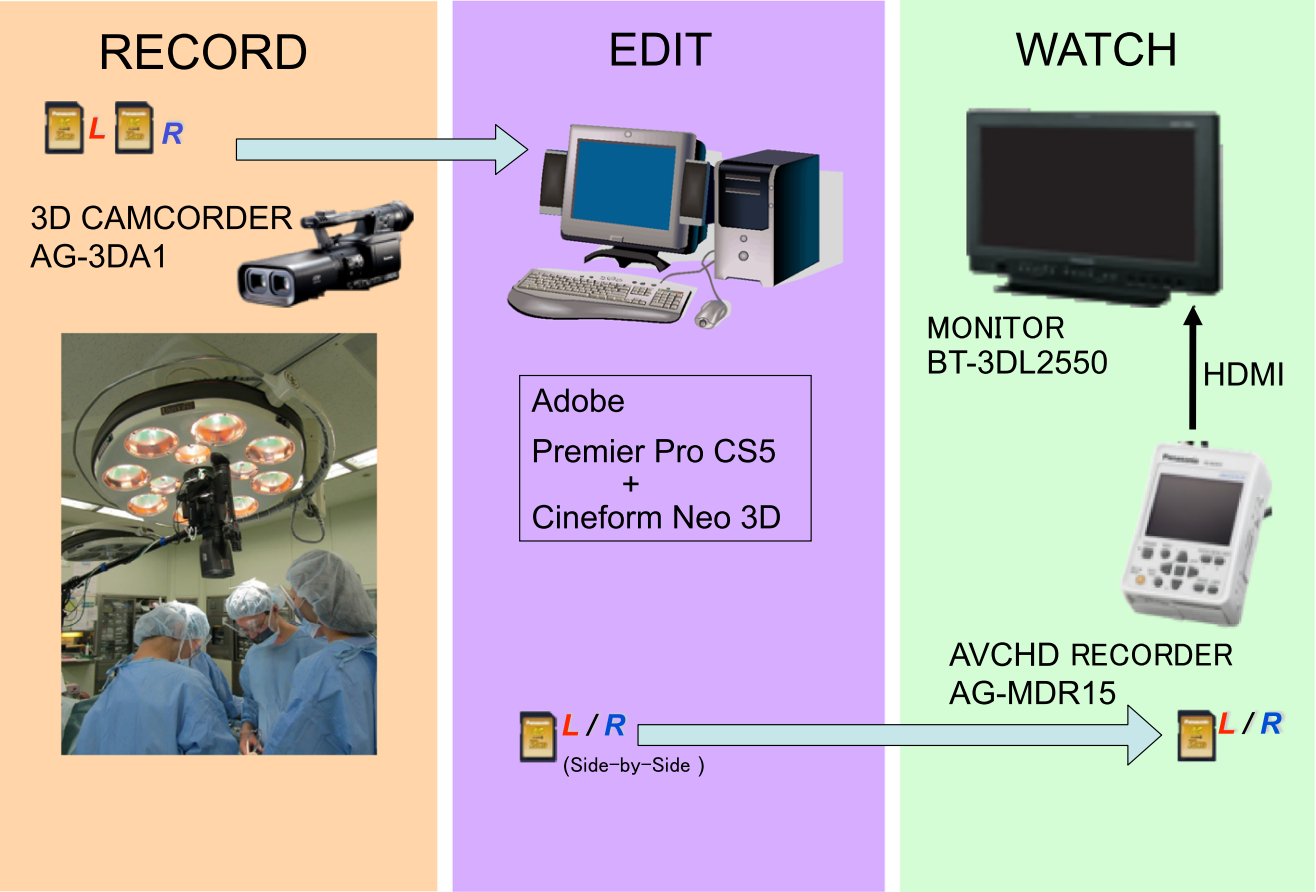
Flowchart of the 3DHD movie recording, editing, and watching

A movie of orthognathic surgery with sagittal splitting ramus osteotomy was recorded using a portable 3D full-HD camcorder (AG-3DA1, Panasonic, Osaka, Japan) and then edited using PC software (Premier Pro CS5, Adove + Cineform Neo 3D, Orbit Musetechs) to be of practical use for education (Fig. 1).

The subjects of this investigation were medical students and doctors receiving surgical training who did not have actual surgical experience (*n* = 65, mean age 26.6 years, age range 23–40 years).

### 1. Viewing the movie

Participants first watched an 8-min, 2D movie of the surgical procedure. They subsequently watched the 3D version of the movie on an 18-inch high-resolution monitor (BT-3DT 2550, Panasonic) while wearing 3D glasses. After watching the 3D movie, participants were asked to complete a questionnaire (Table 1).

**Table 1:** The contents of the questionnaire

Q1: Have you ever watched a 3D movie?	
1) First time, 2) 2–3 times, 3) 4–5 times, 4) More than 6 times	
Q2: How do you feel after watching a 3D movie?	
1) Excellent, 2) Good, 3) Not bad, 4) Bad	
Q3: What do you think the advantages of a 3D movie are?	
1) Appearance of solidity, 2) Realism, 3) Innovation, 4) Other	
Q4: Do you think 3D movies are useful for medical practice?	
1) Quite useful, 2) Useful, 3) Not useful at all	
Q5: What part of medical training or practice would benefit most from 3D movies?	
1) Pre-graduate education, 2) Post-graduate education	
3) Professional education, 4) Explanations of treatment	
5) Research	
Q6: Please write any additional comments.	

### 2. Questionnaire

The contents of the questionnaire were as follows:

Q1: Have you ever watched a 3D movie?

1) First time, 2) 2–3 times, 3) 4–5 times, 4) More than 6 times

Q2: How do you feel after watching a 3D movie?

1) Excellent, 2) Good, 3) Not bad, 4) Bad

Q3: What do you think the advantages of a 3D movie are?

1) Appearance of solidity, 2) Realism, 3) Innovation, 4) Other

Q4: Do you think 3D movies are useful for medical practice?

1) Quite useful, 2) Useful, 3) Not useful at all

Q5: What part of medical training or practice would benefit most from 3D movies?

1) Pre-graduate education, 2) Post-graduate education, 3) Professional education, 4) Explanations of treatment, 5) Research

Q6: Please write any additional comments.

## Results

1. Almost half of participants (45%) had watched 3D movie for the first time in our study, but a 30% of all had watched 2–3 times and 18% of all had watched 4–5 times (Fig. 2).

**Fig. 2:**
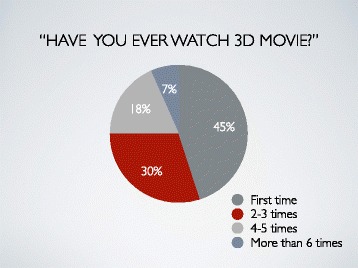
Result 1. Almost half of participants (45%) had watched 3D movie for the first time in our study, but a 30% of all had watched 2–3 times and 18% of all had watched 4–5 times
2. A lot of participants (84%) felt a 3D movie excellent or good. But a little person (2%) had a bad feeling after watching (Fig. 3).

**Fig. 3:**
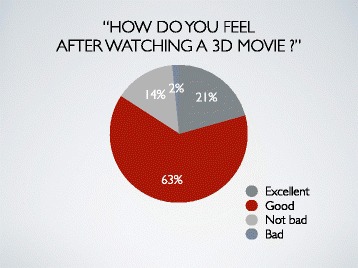
Result 2. A lot of participants (84%) felt a 3D movie excellent or good. But a little person (2%) had bad feeling after watching
3. Many participants answered that the advantages of 3D movie were their appearance of solidity (63%) or realism (25%). An 8% of all answered it was their innovation (Fig. 4).

**Fig. 4:**
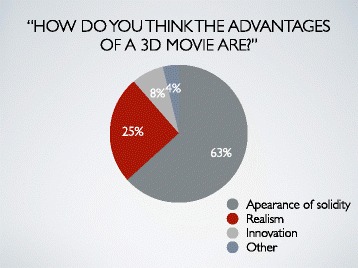
Result 3. Many participants answered that the advantages of 3D movie were their appearance of solidity (63%) or realism (25%). An 8% of all answered it was their innovation
4. Almost all participants (99%) answered that 3D movies were quite useful or useful for medical practice (Fig. 5).

**Fig. 5:**
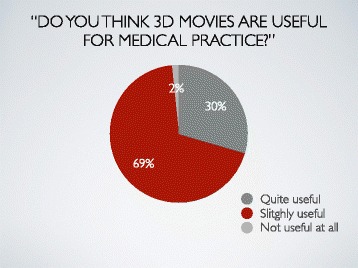
Result 4. Almost all participants (99%) answered that 3D movies were quite useful or useful for medical practice
5. The answers about beneficial parts of medical training or practice from 3D movies were pre-graduate education (20%), post-graduate education (24%), professional education (27%), explanations of treatment (23%), and research (6%) (Fig. 6).

**Fig. 6:**
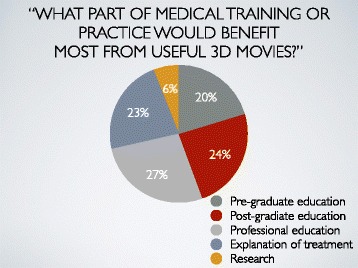
Result 5. The answers about beneficial parts of medical training or practice from 3D movies were pre-graduate education (20%), post-graduate education (24%), professional education (27%), explanations of treatment (23%) and research (6%)
6. Their additional comments were as follows:

“The image quality is excellent, and tint, brightness and vividness are very good, even on a video monitor.” “It is easy to watch and to understand because of the HD quality and 3D depth of the surgical field.” “This system projects a detailed picture and stereoscopic effect, and there is a powerful feeling.” “It is easy to watch the sutures and it is very realistic.” “For educational purposes, there is a realness to it, and the understanding of trainees will be deepened.” “It is easy to understand the three-dimensional aspect of the lesion because of the excellent image quality. It will maintain the view of the operation and contribute to education.” “The depth of the surgical field seems deeper than it actually is when looking at it with my eyes.” “The whole visual field is expanded, but the details are clear.” “Surgical staff will be able to understand the procedure easily.” “If this technology advances, it will be useful for operations with a microscope and endoscope.” “I suffered from motion sickness.” “Because I am not used to watching 3D movies, I felt tired after watching it.”

## Discussion

Conventional medical training required students to read textbooks and take notes. Young doctors usually assisted their older mentors during surgery to study surgical techniques. Nowadays, even in the field of oral and maxillofacial surgery, basic surgical training is shifting from reading textbooks to reading online references [1, 2]. Computerized surgical simulations and plans are already available for some procedures. Additionally, some procedures are now being performed using robot-assisted surgical systems.

In the past, many students watched operations but were unable to see the surgical operative fields well or observe the methods directly. Now, students can watch the surgeries on video monitors in the operating rooms or in distant conference rooms [3, 4].

There are some studies about 3D animation for medical education [5, 6]. But there are few studies about medical 3D movie [11]. We used a prototype 3D full-HD camcorder to record movies of intra-oral surgeries for the first time. In the present study, we assessed the usefulness and capability of 3D full-HD movies for surgical education. Current 3D systems are large and require separate recorders. This camcorder, however, automatically recalibrates without the need for any external equipment, allowing immediate 3D image capture. This camcorder is the world’s first professional twin-lens camera with lenses, camera head, and a dual memory card recorder all integrated into a single, lightweight body.

Originally, anaglyph glasses with separate red and blue lenses were used to generate the stereoscopic 3D effect for movies. When wearing the glasses, part of the 3D image is displayed to the one eye and the second part to the other eye. The modern version of these glasses uses circular polarization. The objection to this technology is that the brightness of the image is essentially halved. Active shutter glasses are used for watching most 3D televisions. The media is displayed at a high frame-rate, and the glasses rapidly switch between opaque and clear using a pair of low-latency transparent liquid-crystal displays. For auto-stereoscopic displays, the lenticular lens method (a saw-tooth prism) was used in the 1990s for “holographic” displays on fancy jewel cases for compact discs. However, there is a relatively small “sweet spot,” and the effective resolution and brightness are halved. To overcome these drawbacks, the screens have to be modified.

After watching the movies, most participants were impressed with the 3D full-HD movie of orthognathic surgery and expressed that it had greater realism than current 2D standard-definition movies. Participants also expressed that the system would be very helpful for their medical practice and surgical training. From the opinions about the 3D full-HD movie, advantages included the quality of the image, fineness, and enhanced realism due to the depth and dynamics of the images (Table 2). Regarding the possibilities of 3D full-HD movies, some participants expected them to have a role in medical education and to deepen the understanding of medical practice [12, 13].

**Table 2:** Advantages and disadvantages of 3DHD movie

Advantages	
• Enhanced realism due to the solidity and dynamics.	
• The quality of the image and fineness.	
• Could be applied to surgical education and medical practice.	
Disadvantages	
• 3D camcorder, 3D monitor, and special glasses are required.	
• The current system is expensive.	
• Someone feels motion sickness.	

With technological advancements, one participant commented that 3D full-HD movies could be applied to microsurgeries and endoscopic operations.

As objections to the technology, some participants felt that the depth of field was deeper than what would be expected naturally, and others who were not used to virtual 3D suffered from motion sickness [14]. Special glasses are required to watch the current 3D full-HD movie, and several participants responded that they hope for improvement about glass-free watching [15].

Three-dimensional full-HD movies will be useful for surgical simulations, medical education, training, and explanation of procedures or findings to patients, and for 3D simulators. However, the current 3D movie system is expensive and requires a 3D camcorder and 3D monitor. There are also some issues regarding motion sickness and the realism of the 3D depth.

When movies of medical procedures were recorded over half a century ago, it must have seemed epoch-making. However, movies are a crucial part of any medical field today [16, 17]. While the use of medical 3D full-HD movies is not yet widespread, proliferation of this technology is expected in the future.

## Conclusions

Three-dimensional full-HD movies have the potential to improve the quality of medical education and clinical practice in oral and maxillofacial surgery. But there are some issues regarding motion sickness and the realism of the 3D depth.
