# Erratum to: Usefulness and capability of three-dimensional, full high-definition movies for surgical education

**DOI:** 10.1186/s40902-017-0124-2

**Published:** 2017-08-16

**Authors:** M. Takano, K. Kasahara, K. Sugahara, A. Watanabe, S. Yoshida, T. Shibahara

**Affiliations:** 1 Department of Oral and Maxillaofacial Surgery, Tokyo Dental College, 101-0061, 2-9-18 Misakicho, Chiyoda-ku, Tokyo, Japan; 2 Department of Oral Pathobiological Science and Surgery, Tokyo Dental College, Mihama-ku, Japan

## Erratum

In the publication of this article [1], there is an error in the Reference section at reference 16.

The error: ‘de Heer Maier H. G, Ortac A, Kuijten J (2015) Capturing and displaying microscopic images used in medical diagnostics and forensic science using 4K video resolution—an application in higher education. J Microsc 260:175–179’ Should instead read: ‘Maier H. de Heer, G, Ortac A, Kuijten J (2015) Capturing and displaying microscopic images used in medical diagnostics and forensic science using 4K video resolution—an application in higher education. J Microsc 260:175–179’. This has now been included in this erratum.
